# Supplementary material for: Effect of Yeast Saccharomyces cerevisiae as a Probiotic on Diet Digestibility, Fermentative Metabolites, and Composition and Functional Potential of the Fecal Microbiota of Dogs Submitted to an Abrupt Dietary Change
Source: Microorganisms. 2023 Feb 17;11(2):506. doi: 10.3390/microorganisms11020506 (PMC9965016; doi:10.3390/microorganisms11020506)
Supplement: Supplementary file 1 [file microorganisms-11-00506-s001.zip › microorganisms-2110671-supplementary.pdf]

Supp. Table S1. Means of the relative abundance (%) of bacterial genera that statistically differed in feces of dogs fed the lower protein and fiber diet (LPF, day 21) transitioned to the higher protein and fiber diet (HPF, days 23 to 49) without (C) or with (P) yeast supplementation (S).

Item	S	Day (D)					SEM	*P-values*		
		0	21	23	35	49		D	S	D×S
Streptococcus	C	21.6^{b}	43.8^{a}	37.8^{a}	0.5^{c}	0.1^{c}	5.2	<0.001	0.215	0.831
	P	28.6^{b}	56.3^{a}	43.8^{a}	0.1^{c}	0.0^{c}	5.7			
Lactobacillus	C	40.4^{a}	40.1^{a}	31.2^{b}	3.8^{c}	15.9^{c}	4	<0.001	<0.001	0.363
	P	18.9^{a}	15.2^{a}	4.3^{b}	1.2^{c}	1.7^{c}	3.1			
Collinsella	C	14.8^{a}	4.4^{b}	11.5^{a}	29.6^{a}	23.4^{a}	2.1	0.003	0.574	0.187
	P	23.8^{a}	9.1^{b}	21.1^{a}	24.5^{a}	13.9^{a}	2			
Bifidobacterium	C	1.9^{b}	6.1^{a}	1.8^{b}	0.7^{b*}	8.8^{a*}	3.5	<0.001	<0.001	<0.001
	P	0.6^{c}	11.5^{b}	3.8^{bc}	39.1^{a*}	52.2^{a*}	4.7			
Blautia	C	2.3^{b}	0.4^{c}	1.4^{b}	5.7^{a}	9.8^{a}	0.7	<0.001	0.237	0.056
	P	1.7^{b}	0.7^{c}	3.1^{b}	5.0^{a}	4.9^{a}	0.6			
Allobaculum	C	0.8^{b}	0.3^{c}	2.6^{ab}	6.6^{a}	7.6^{a}	0.4	<0.001	0.488	0.139
	P	2.1^{b}	0.7^{c}	3.8^{ab}	4.9^{a}	4.1^{a}	0.6			
Lachnospiraceae	C	5.2^{a}	0.3^{b}	0.9^{ab}	5.3^{a}	4.7^{a}	0.5	<0.001	0.253	0.203
	P	3.0^{a}	0.8^{b}	2.7^{ab}	3.4^{a}	3.0^{a}	0.4			
Turicibacter	C	3.7^{a}	0.1^{c}	0.9^{bc}	2.0^{b}	2.7^{b}	0.4	<0.001	0.029	0.394
	P	7.8^{a}	1.4^{c}	2.2^{bc}	2.5^{b}	2.9^{b}	0.5			
Romboutsia	C	0.8^{a}	0.0^{b}	0.3^{b}	1.6^{a}	1.6^{a}	0.2	<0.001	0.901	0.431
	P	1.6^{a}	0.1^{b}	0.4^{b}	1.1^{a}	1.2^{a}	0.2			
Holdemanella	C	0.2^{b}	0.2^{b}	0.7^{ab}	2.0^{a}	1.3^{a}	0.1	<0.001	0.556	0.455
	P	0.2^{b}	0.3^{b}	1.0^{ab}	1.3^{a}	1.1^{a}	0.1			
Catenibacterium	C	0.1^{c}	0.1^{c}	0.4^{ab}	1.6^{a}	1.7^{a}	0.2	<0.001	0.358	0.184
	P	0.1^{c}	0.3^{c}	0.7^{ab}	1.1^{a}	1.1^{a}	0.1			
Clostridiales	C	0.6^{ab}	0.1^{b}	0.3^{ab}	1.2^{a}	1.1^{a}	0.1	<0.001	0.667	0.434
	P	0.5^{ab}	0.2^{b}	0.6^{ab}	0.9^{a}	0.7^{a}	0.1			
Clostridium	C	0.8^{a}	0.0^{b}	0.4^{b}	0.5^{b}	0.3^{b}	0.1	<0.001	0.963	0.347
	P	1.3^{a}	0.0^{b}	0.1^{b}	0.4^{b}	0.3^{b}	0.1			
Enterococcus	C	0.5^{a}	0.0^{b}	0.0^{b}	0.0^{b}	0.0^{b}	0.0	<0.001	0.779	0.829
	P	0.3^{a}	0.0^{b}	0.1^{b}	0.0^{b}	0.0^{b}	0.0			
Lachnoclostridium	C	0.1^{a}	0.0^{b}	0.1^{a}	0.2^{a}	0.2^{a}	0.0	<0.001	0.247	0.352
	P	0.1^{a}	0.0^{b}	0.1^{a}	0.1^{a}	0.1^{a}	0.0			

^{a,b}Different superscript letters indicate differences among days by the LSD Bonferroni test in each group ($P<0.05$ for D×S) or for both groups ($P>0.05$ for D×S). *Indicate difference between Control and Probiotic groups at the time point.

SEM: standard error of the mean.

Supp. Table S2. Means of the relative abundance (%) of the most abundant bacterial species that statistically differed in feces of dogs fed the lower protein and fiber diet (LPF, day 21) transitioned to the higher protein and fiber diet (HPF, days 23 to 49) without (C) or with (P) yeast supplementation (S).

Item	S	Day (D)					SEM	*P-values*		
		0	21	23	35	49		D	S	D×S
Lactobacillus animalis	C	34.7^{a}	33.0^{a}	20.7^{b}	12.5^{c}	10.9^{c}	3.9	0.011	<0.001	0.897
	P	15.9^{a}	13.8^{a}	7.3^{b}	0.7^{c}	3.1^{c}	2.0			
Bifidobacterium pseudolongum	C	1.4^{c}	4.4^{b}	4.2^{b}	3.2^{bc*}	9.1^{a*}	1.6	<0.001	<0.001	0.002
	P	0.6^{c}	10.7^{b}	3.4^{c}	36.2^{a*}	45.4^{a*}	4.3			
Collinsella intestinalis	C	12.6^{a}	3.4^{b}	9.2^{a}	27.8^{a}	22.0^{a}	2.3	0.001	0.681	0.204
	P	20.9^{a}	7.4^{b}	17.1^{a}	22.8^{a}	12.7^{a}	2.2			
Streptococcus lutetiensis	C	3.3^{b}	6.7^{a}	5.8^{ab}	0.1^{c}	0.1^{c}	0.6	<0.001	0.206	0.838
	P	4.6^{b}	8.5^{a}	6.6^{ab}	0.0^{c}	0.0^{c}	0.7			
Clostridium hiranonis	C	1.6^{b}	0.4^{c}	1.3^{b}	4.3^{a}	3.4^{b}	0.3	<0.001	0.018	0.172
	P	2.9^{b}	1.1^{c}	3.5^{b}	6.8^{a}	2.5^{b}	0.4			
Turicibacter sanguinis	C	1.3^{a}	0.2^{b}	0.4^{b}	1.4^{a}	1.2^{a}	0.2	0.009	0.048	0.726
	P	2.8^{a}	0.6^{b}	0.9^{b}	2.0^{a}	1.3^{a}	0.3			
Blautia hansenii	C	0.8^{a}	0.1^{b}	0.1^{b}	0.3^{b}	0.3^{b}	0.1	0.009	0.638	0.927
	P	1.1^{a}	0.1^{b}	0.2^{b}	0.2^{b}	0.2^{b}	0.1			

^{a,b}Different superscript letters indicate differences among days by the LSD Bonferroni test in each group (P<0.05 for D×S) or for both groups (P>0.05 for D×S). *Indicate difference between Control and Probiotic groups at the time point.

SEM: standard error of the mean.

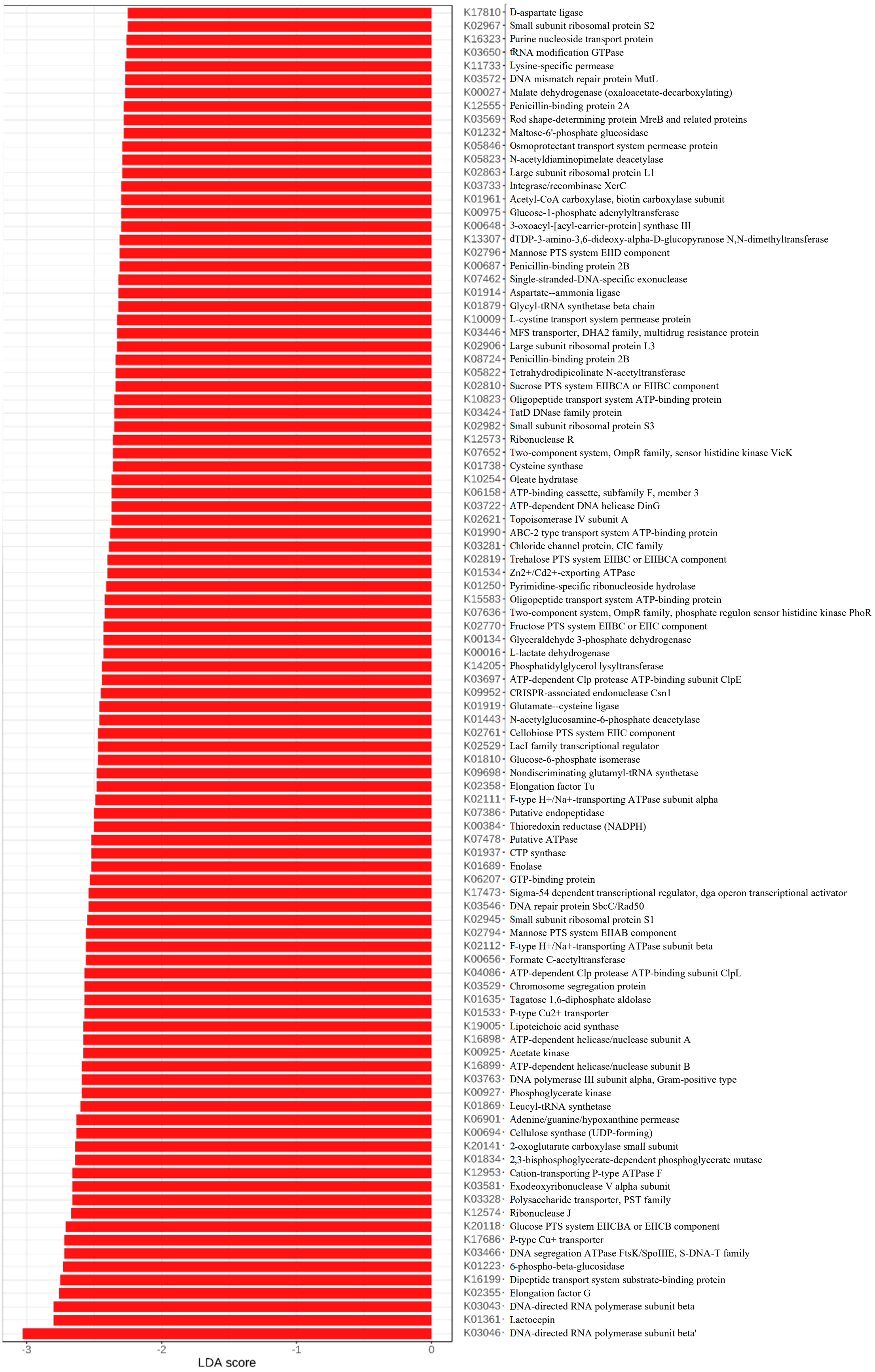

Supp. Figure S1. LDA score of KO terms that were enriched only in the lower protein and fiber diet – day 21 (red) in comparison to the higher protein and fiber diet – day 49 (P<0.05).

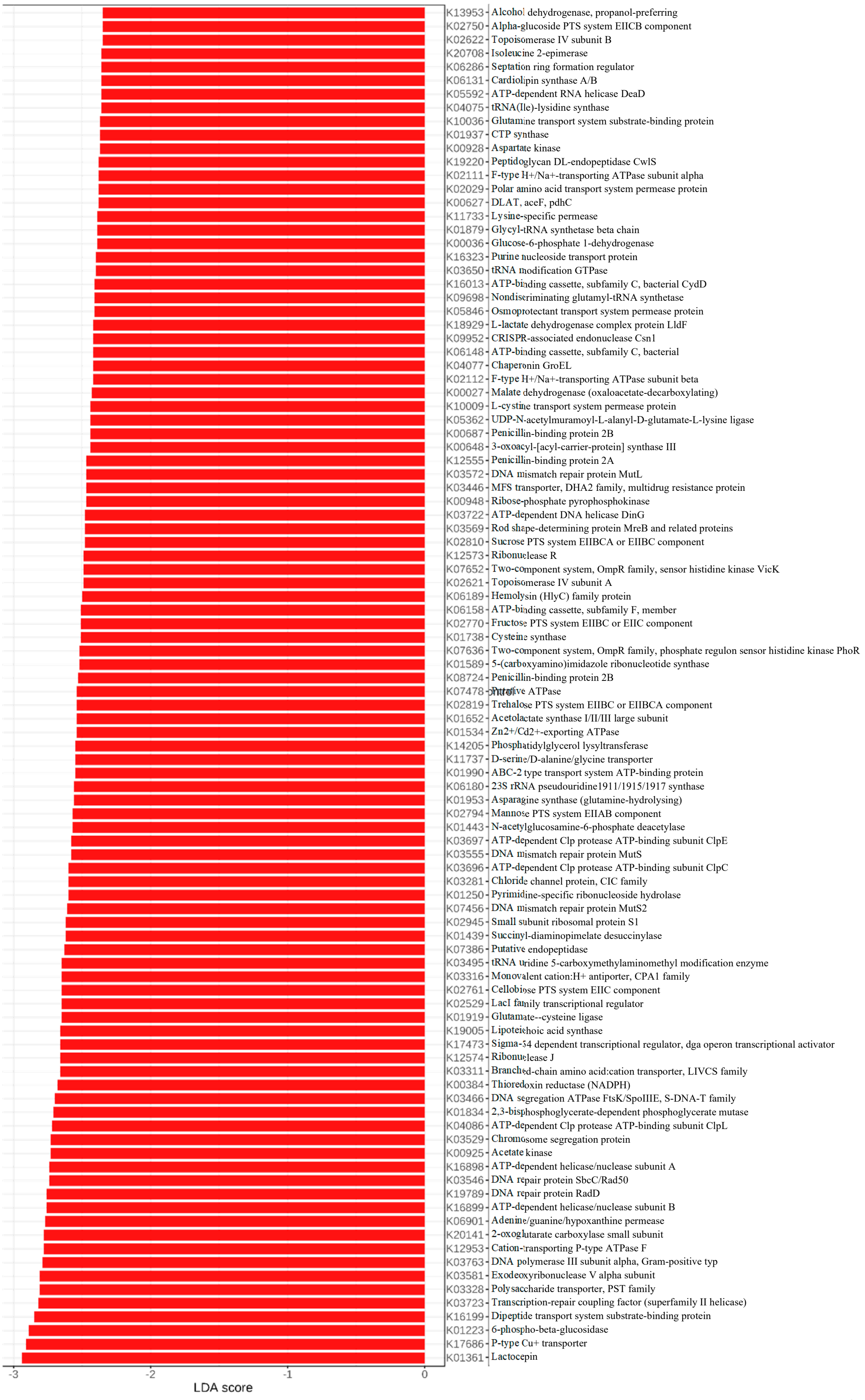

Supp. Figure S2. LDA score of KO terms that were enriched only in the control group (red) in comparison to the probiotic group fed the lower protein and fiber diet – day 21 (P<0.05).

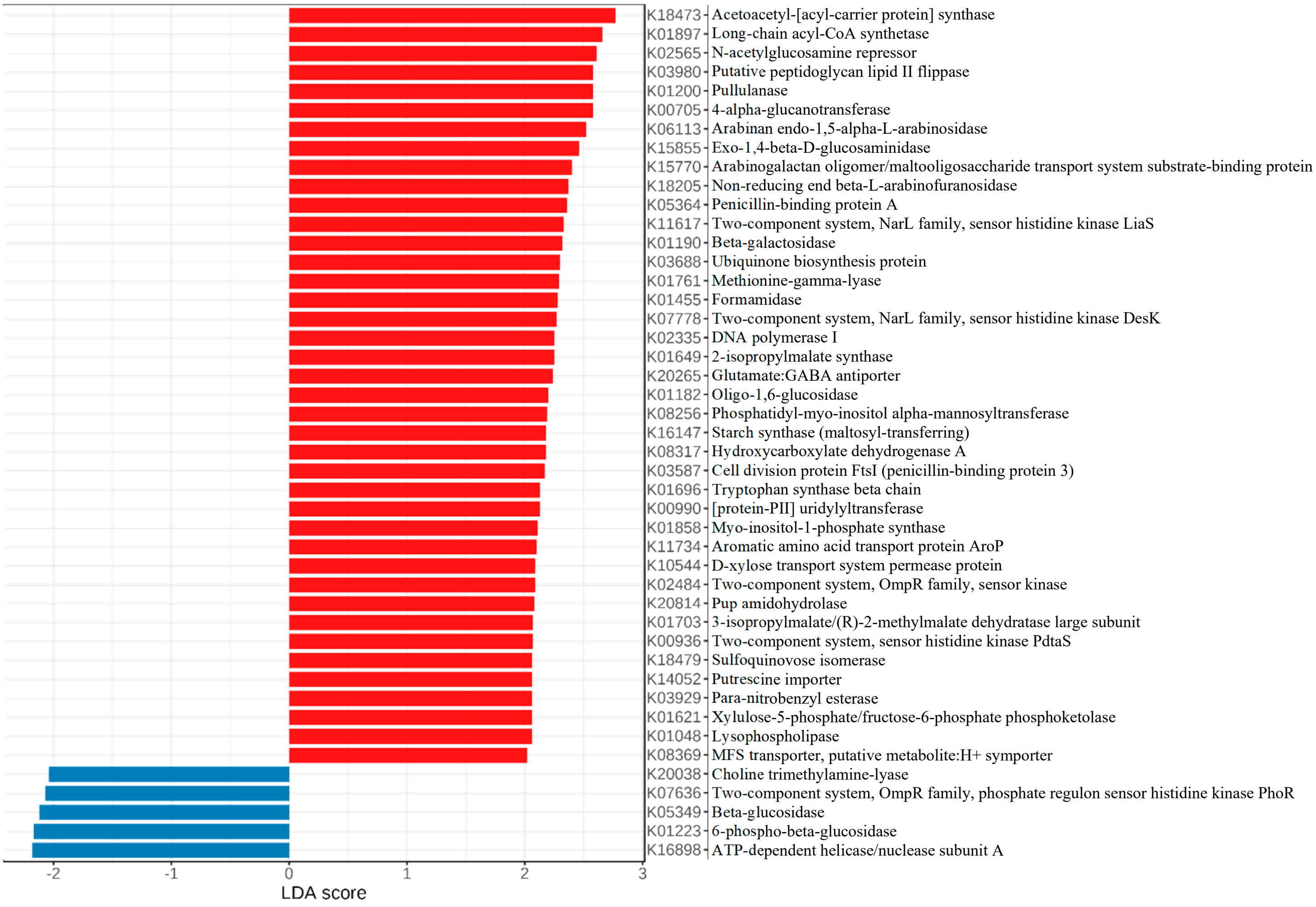

Supp. Figure S3. LDA score of KO terms that differ between the control (blue) and probiotic (red) groups fed the higher protein and fiber diet - day 49 (P<0.05).
